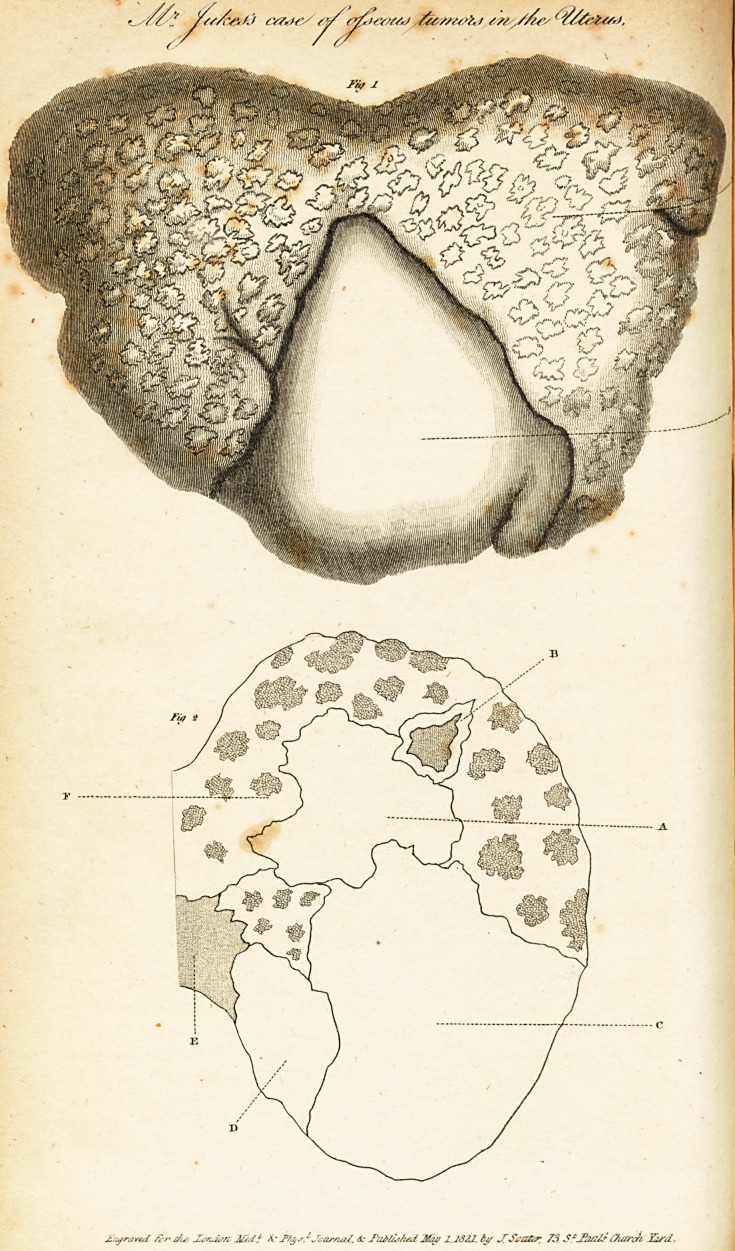# An Account of Some Osseous Tumors Found in the Uterus of a Woman

**Published:** 1821-05

**Authors:** Edward Jukes

**Affiliations:** Surgeon, Accoucheur to the Westminster Medical Institution and Lying-in Charity.


### Mr. Jukes's case of osseous tumors in the Uterus.

Fig 1

Fig 2

Engraved for the London Medl. & Physl. Journal, & Published May 1. 1821. by J. Souter, 73. St. Paul's Church Yard.

THE LONDON

### Medical and Physical Journal.

### Original Communications, Select Observations, etc.

*An Account of some Osseous Tumors found in the Uterus of a Woman.* By Mr. EDWARD JUKES, Surgeon, Accoucheur to the Westminster Medical Institution and Lying-in Charity.

[With an Engraving.]

ON examining, a few days since, the body of a woman, aged 67, who, as it appeared, died in consequence of chronic inflammation of the mucous membrane of the stomach and small intestines, I found the uterus present the following remarkable appearances. It was of the size of the head of a fetus of seven months, of a deep and dullish red colour externally; and the trunks of its principal blood-vessels were much increased in volume. The ovaria differed in colour from the uterus, having nearly the ordinary hue. The right one presented several points resembling the cicatrices formed after the escape of ova; in the left, only one mark of this sort was perceptible. Two vesicles projected from the surface of the right ovarium; one of them of the size of a lupin-seed, the other of that of a small pea: these vesicles, the coats of which were very thin and transparent, contained a yellowish fluid, which coagulated by heat. The cavity of these vesicles could be traced to only a small extent beneath the surface of the ovarium: their situation may be very well described, by stating that they appeared like vesicles which had been arrested just as they were on the point of total separation from the ovarium. Both ovaria contained several corpora lutea. The fallopian tubes were in the ordinary state, excepting that they partook of the inordinate vascular appearance of the uterus. The os uteri was much thickened, though without the least inordinate hardness: its orifice was of about the size of a straw, and its margin very sharp. A canal, of nearly the same diameter as this orifice, extended through the neck of the uterus to a cavity of larger dimensions, which appeared to have been the proper cavity of the uterus. The structure of this organ was like a very dense cellular tissue, of a deep-red colour throughout, and about half an inch in thickness at the neck and the lower part of the body of the uterus; about the fundus it varied much, from the causes which will be presently described. Not the slightest semblance of muscular structure, or regular fibrous arrangement, could be discerned. Four distinct tumors projected from the exterior surface of the uterus: one, of about the bulk of a walnut, about the middle of the fundus; two, somewhat smaller, a little below it, posteriorly; and one at the anterior part of the uterus near the fundus. These tumors were seated in the midst of the parietes of the uterus; their surface was moderately smooth, and adherent to the surrounding soft parts: they appeared to have been enveloped in proper cysts, or a dense membrane had been formed around them by the adjacent cellular texture. One of them, on being divided by a saw, was found to be constituted of a very firm osseous mass, more compact towards the surface than in the centre, where it presented a cellular appearance, like that of the heads of the long bones in an adult. It had no distinct central cavity. The other tumors were not opened; but they seem to be of a similar kind.

On dividing what appeared, externally, to be the uterus, by a longitudinal incision from the fundus to the neck, the greater part of the mass was found to be formed by a large osseous tumor, which at first seemed to have been seated in the natural cavity of the uterus; but, on more minute examination, it was found that it was distinct from this cavity, and situate in the midst of the parietes at the fundus. This tumor was enveloped in a very dense cyst, by which it adhered to the surrounding soft parts: its surface was very irregular, being studded throughout, except at the somewhat distinct portion, A, fig. 1, in the annexed Plate, with small, rough, osseous protuberances; which were, however, covered by the cyst enveloping the tumor generally. The capsular membrane of the somewhat distinct portion, (thus A. referred to in the Plate, fig. 1,) was much firmer than that of the rest of the tumor, and presented a smooth polished surface. This was the most inferior part of the tumor, and was situate just over the cavity of the uterus.

The size of this tumor was that of a man's fist: its dimensions, as well as its shape, are accurately shown by the figures in the annexed Plates. Figure 1, represents the exterior appearance of the tumor; B, is the part of the surface studded with the bony protuberances; A, is a somewhat distinct portion of the tumor, separate from the rest to the distance of three or four lines below the surface. On the tumor being sawed through the middle, and the two portions turned aside, the severed surface of one of which is shown in Figure 2, the greater portion of it, F. was found to be constituted of a dense cellular texture, interspersed with bony masses of various dimensions, from a portion of three inches in circumference, (A.) to others not larger than a pea. Some portions (as in B.) were constituted interiorly of a dense cellular substance; whilst others, and the greater number, were formed throughout of a mass of bone arranged in a cellular structure, somewhat like the heads of the long bones of an adult. C. fig. 2, show the interior distribution of the somewhat distinct portion.—A. fig. 1. This was constituted chiefly of a very dense cellular tissue, almost as firm as cartilage, having small osseous tubercles dispersed in it only here and there.—D. is a sort of subdivision of this distinct portion of the tumor, which was parted from the rest by a line of cellular substance: this subdivision was constituted of very dense cellular, or cartilaginous, substance, without ossification. —E. is another nearly distinct portion, formed of a more loose cellular texture.

The weight of the whole tumor was seventeen ounces.

There were no other remarkable morbid or preternatural appearances in the body than those just described, and such as were indicative of chronic inflammation of the mucous membrane of the stomach and intestines. This membrane was throughout studded with dark brownish-red patches, where it was much thickened, and in several parts disorganized: but, as these appearances do not appear to bear any particular relation to the tumor in question, I need not minutely detail them.

I did not see the subject of this disease until just the instant after her death, and I have not been able to collect any very particular information respecting her state of health and habits. There were no persons about her who knew much of her private life; nor have I been able to find any of her family relations. All I can learn, is, that she had been thrice married, —first, at some period before the twentieth year of her age; that she never had a child; and that her health had been tolerably good, until of late, when she experienced disorder of the stomach, which was attributed to the excessive quantity of gin she was in the daily habit of drinking. Her neighbours did not know that she had, for several years past, received any medical aid; and it does not appear that she ever complained, to persons about her, of any symptoms referrible to the disease of the uterus.

The recorded instances of osseous tumors seated in the cavity or the parietes of the uterus, are by no means rare:* still I think the example of disease above related presents some circumstances which render it worthy of publication. It seemed to be particularly interesting to Mr. ASTLEY COOPER,† on my submitting it to his inspection; and this consideration alone would have been sufficient to induce me to offer the foregoing account for insertion in the Medical and Physical Journal.

*London; Feb.* 20, 1821.

* Examples may be found in LIEUTAUD, (*Hist. Anat. Med.* tom. i.;) the *Memoires de l'Acad. de Chirurgie,* tom. ii.; the *Philosophical Transactions,* 1733; *London Med. and Phys. Journal,* vol. iii.; the *Ephemerides Natur. Curios.*; PARÉ, BARTHOLINUS, MORUS, and even in the writings attributed to HIPPOCRATES: though the precise nature of the whole of the cases related by the authors here referred to, is not clearly evident; as the older writers appear to have confounded osseous tumors with calculous formations, describing both under the latter appellation.

† It may not be improper for me to remark, that the preparation is now in the possession of Mr. Astley Cooper.

## Figures and Tables

**Fig 1 Fig 2 f1:**